# Myasthenia gravis and schizophrenia co-morbidity: psychiatric treatment challenges, a case report

**DOI:** 10.1192/j.eurpsy.2026.11859

**Published:** 2026-07-31

**Authors:** I. Mosbah, H. Ghabi, M. Mouldi, H. Nefzi, R. Kammoun, M. Karoui, F. Ellouze

**Affiliations:** 1 Department of Psychiatry G, Razi hospital, Manouba; 2 El Manar University, Faculty of Medicine of Tunis, Tunis, Tunisia

## Abstract

**Introduction:**

Myasthenia gravis (MG) is a rare autoimmune disorder caused by autoantibodies targeting the neuromuscular junction, with an estimated prevalence of fewer than 10 per 100,000 individuals. Schizophrenia is a psychiatric disorder that affects approximately 1% of the population. The coexistence of MG and schizophrenia is exceptionally uncommon, with only a few cases reported. This combination poses significant therapeutic challenges, as several antipsychotics possess anticholinergic properties or interfere with neuromuscular transmission, potentially exacerbating MG symptoms.

**Objectives:**

To describe a rare case of comorbid myasthenia gravis and schizophrenia, highlighting the challenges of antipsychotic management and the importance of individualized, collaborative care.

**Methods:**

We present the case of a 61-year-old woman with schizophrenia who subsequently developed MG. The clinical course, therapeutic interventions, and outcomes were closely monitored under the joint care of psychiatry and neurology teams.

**Results:**

The patient had been treated in our department since 2000 for schizophrenia and was initially stabilized on haloperidol decanoate (100 mg monthly injection) due to poor adherence to oral medication. In 2009, she developed muscular weakness and dyspnea, which led to a diagnosis of myasthenia gravis and initiation of acetylcholinesterase inhibitors. Haloperidol was temporarily discontinued due to safety concerns, and trials of risperidone (2 mg) and olanzapine (10 mg), introduced with slow titration, were attempted. However, psychiatric symptoms worsened, and myasthenic manifestations emerged under risperidone. Reintroduction of haloperidol (7.5 mg) resulted in significant improvement in psychotic symptoms, including a reduction of delusions and hallucinations, without clinical deterioration of MG.

**Conclusions:**

Managing schizophrenia in patients with MG is challenging due to limited guidance and potential drug interactions. Our case demonstrates that haloperidol can be effective and well tolerated, emphasizing the need for careful antipsychotic selection, close monitoring, and multidisciplinary collaboration in such rare comorbid presentations

**Disclosure of Interest:**

None Declared

